# Millimeter-wave multi-mode circular antenna array for uni-cast multi-cast and OAM communication

**DOI:** 10.1038/s41598-021-83301-1

**Published:** 2021-03-02

**Authors:** Stylianos D. Assimonis, Muhammad Ali Babar Abbasi, Vincent Fusco

**Affiliations:** School of Electronics, Electrical Engineering and Computer Science, Queen’s University Belfast, Belfast, BT3 9DT UK

**Keywords:** Engineering, Electrical and electronic engineering

## Abstract

This paper investigates uni-/multi-cast and orbital angular momentum (OAM) mode data transmission in orthogonal directions by the utilization of a new circular antenna array, operating at 28 GHz. In the horizontal plane the proposed antenna array operates as multimode transmitter (i.e., it provides broad-, uni- and/or multi-cast communication), while in the vertical direction OAM transmission occurs (i.e., it is capable of generating up to 15 spatially orthogonal OAM modes). Antenna array is designed using twelve, low-complexity, electromagnetically coupled microstrip patch antennas with high radiation efficiency. Each of these can transmit power of equivalent order of magnitude in both horizontal (i.e., broadside radiation pattern) and vertical direction (i.e., endfire radiation pattern) over electromagnetic waves of orthogonal electric components. This property leads to the formation of uni-/multi-cast and OAM modes in the horizontal plane and vertical direction, respectively. Antenna was tested through full-wave electromagnetic analysis and measurements in terms of impedance matching, mutual coupling and radiation pattern: good agreement between simulated and measurement results was observed. Specifically, it presents up to 8.65 dBi and 6.48 dBi realized gain under the uni-cast (in the horizontal plane) and OAM mode (in the vertical plane), respectively. The proposed antenna array is perfect candidate for high spectral efficiency data transmission for 5G and beyond wireless applications, where orthogonality in communication links and OAM multiplexing is a requirement.

## Introduction

The development of wireless multimedia communication techniques over the last years requires the design of new, high-directive^1^, compact^2^, reconfigurable^3^, multiple-input–multiple-output (MIMO)^4^ and meta-surface antenna systems^5^. At the same time, due to congestion in the radio frequency spectrum below 10 GHz, the fifth generation of wireless communication (5G) is now exploring the best possible options at the millimeter-wave (mmWave) spectrum. From the wealth of literature on communication system hardware used for the sub-6 GHz 5G, 4G and earlier generations of cellular communication, Global System for Mobile Communications (GSM), Long Term Evolution (LTE) and wireless fidelity (Wi-Fi), we can easily envision that the most valuable resource in the future will be the efficient use of the spectrum. New disruptive technologies are currently being explored in the communication system community to preserve the spectrum as much as possible. Multimode circular array and Orbital Angular Momentum (OAM) transmitters are a few such technologies. Compared to standard radio, the mode-based circular array radiation is promising in terms of spectrum efficiency^6^, since it can be used for orthogonal data transmission by utilizing, at the same time, spatial and frequency resources.

Wireless channel is inherently a broadcast medium, while, uni- and multi-cast radio transmission imply that a conventional wireless channel can be converted into a beam-space channel^7,8^. Typically, the multi-cast technique is employed by routing at the network layer. Uni- and multi-cast techniques at physical layer mean that the array equipped at the base-station (BS) or access point is creating suitable beam-patterns to serve single or multiple user groups simultaneously using same bandwidth. By doing this, significant benefits in terms of spectral efficiency and reduced latency can be achieved. Beam-pattern shaping also means high directivity transmission of radio signal in prescribed directions, which in turn enhances the signal-to-noise-ratio (SNR) at the receiver end^9,10^, increasing the overall communication system efficiency and throughout. Since the channel-state-information (CSI) is only accessible at the physical layer, uni- and multi-cast transmission at the physical layer is a complementary technique to the network-layer multi-cast routing, resulting in enhanced quality of the wireless communication system.

Several studies have investigated novel OAM mode radiation approaches, which use the same time and frequency resources for the data transmission, while the radiation mechanism of OAM is different compared to multi-cast radiation. Some examples of OAM radios are based on reflector-array^11^ metasurfaces^12,13^, microstrip patch antenna^13,14^, helical antenna arrays^15^, and ceramic antenna arrays^16^. Generally, a multimode array is connected to a feed network, which is responsible for generating the magnitude and phase of the antenna feeds required to generate spatially orthogonal modes^17^. OAM wave-fronts can be generated without the requirement of feed networks by specialized antenna geometries like in^16^. Multi-mode feed networks generally contain multiple phase shifters, switches, attenuators or radio frequency (RF) electronic devices that can control the magnitude and phase of the antenna feeding signal. For simpler cases of mode transmission, standard phase delay transmission lines are enough to realize the required antenna feed sequence. Reconfigurability is also included in some of these feed networks so that more sophisticated radio wave propagation modes can be handled by the transmitter^13^. Complex feed structures like the Rotman lens are also shown to be useful for OAM multimode feed networks^18–20^.

In general, a circular antenna array consisting of elements, which radiate power to a specific direction, can form OAM modes and transmit towards this direction. For example, a circular antenna array consisting of patch antenna elements, which lay in parallel to the horizontal plane, is able to form OAM modes, given an appropriate excitation, and transmit power in the vertical direction, e.g.,^18,20^. However, if the patch antenna elements are placed vertically in the horizontal plane, the corresponding circular antenna array cannot transmit OAM modes in the vertical direction, since each of the elements radiates power mainly in the horizontal plane. On the other hand, this antenna array can form uni- and multi-cast modes and transmit power in the horizontal plane. The novelty of this work is the solution of this engineering problem: how to design a circular antenna array, able to form spatially separated uni-/multi-cast and OAM modes. The solution of this problem is the utilization of radiating elements, which transmit power in two orthogonal directions, over two orthogonal electric components. Specifically, the electromagnetic coupled patch element, which have been chosen in this work, it is placed vertically in the horizontal plane, but transmits power in both the horizontal plane, over the electric component $E_{\theta }$ (i.e., electric component in polar $\theta$ direction), and also in the vertical direction, over the electric component $E_{\phi }$ (i.e., electric component in azimuthal $\phi$ direction), as it will be shown. Thus, the proposed circular antenna array, consisting of these elements, is able to produce both uni-/multi-cast modes in the horizontal plane, and also, OAM modes in the vertical direction. Hence, to the best of our knowledge, it is the first antenna that has been designed and fabricated at 28 GHz which presents this double functionality.

Thus, the contribution of this work is two-fold. Firstly, we present a high radiating efficiency 12-element circular array operating at 28 GHz, capable of broadcast, uni-cast and multi-cast radio transmission along the entire $360 ^{\circ }$ azimuth plane. Secondly, we show that the same circular array is capable of generating as many as 15 spatially orthogonal OAM modes in the vertical direction. The antenna array was tested through full-wave electromagnetic analysis and measurements in terms of reflection coefficients, mutual coupling between the array elements and radiation pattern (near- and far-field).

This type of antenna is a perfect candidate for applications where on-demand azimuth and elevation coverage and OAM multiplexing is desirable, e.g., in drone networks. One example of such scenario is the communication between a drone and a base-station (BS), which is based on ground and equipped with the circular antenna array. Drone-to-BS link relies on the OAM modes, while BS-to-devices on ground, e.g., sensor nodes of a wireless sensor network (WSN), relies on the uni- and multi-cast modes. Thus, the proposed antenna serves as WSN-gateway through the drone. Another application is the communication between swarms of connected drones equipped with the circular array, where drone-to-drone communication is established along the horizontal plane, i.e., through the uni- and multi-cast modes, while drone-to-BS, which is now placed on some or a single drone, communication occurs in vertical direction, by utilizing the OAM modes.

This paper is organized as follows. First, we present the synthesis technique, the proposed antenna array and the radiation mechanism of the single antenna element, which is the main pillar of the multimode function of the proposed antenna. Next, the proposed antenna array is analysed via full electromagnetic simulations and measurements in terms of uni-/multi-cast operation, and then, in terms of OAM transmission. A description of the simulation and measurement setup is also given. Finally, this study on the proposed antenna is concluded.

## Results and discussion

### Bi-functional circular antenna array

In this section, the proposed antenna will be theoretically analysed and designed though full-wave electromagnetic analysis. All results will be cross-checked through measurements.

#### Theoretical analysis

The radiation patterns of a circular antenna array with *N* equally spaced elements and radius of *R*, which is placed in the horizontal plane and specifically it centre lies at the origin of coordinates, is given in the classical array literature^21,22^,

1 $$\begin{aligned} E^{\,m} \left( \theta , \phi \right) = \sum \limits _{n=0}^{N-1} \mathbf {w}^{\,m} E_n \, e^{j k R \sin \theta \, \cos \left( \phi -\phi _n \right) } \end{aligned}$$

where,

2 $$\begin{aligned} \phi _n = n \frac{2 \pi }{N}, \end{aligned}$$

is angular spacing between antenna elements, $\theta , \phi$ is the polar, azimuthal angle respectively, $k=2\pi /\lambda$ is the wavenumber and $\lambda$ is the wavelength at the operating frequency, $E_n$ is the single antenna element’s radiation pattern, which is usually assumed that $E_n =1$, indicating isotropic elements, and

3 $$\begin{aligned} \mathbf {w}^{\,m} = \left[ \ldots ,~e^{j m \phi _n},~\ldots \right] ^{T}. \end{aligned}$$

is the vector related to the *m*-th mode, which represents the current excitation on each element, where,

4 $$\begin{aligned} m = 0,~\pm 1,~\ldots ,~\pm \left( \frac{N}{2}-1\right) ,~\frac{N}{2} , \end{aligned}$$

assuming that *N* is even number, without loss of generality. Thus, for an antenna array with *N* elements it is possible to excite *N* modes. In order to steer the beam to a specific direction along the horizontal plane, an extra angle, $\psi$, can be utilized as follows:

5 $$\begin{aligned} \mathbf {w}^{\,m} = \left[ \ldots ,~e^{j m \left( \phi _n + \psi \right) },~\ldots \right] ^{T}. \end{aligned}$$

Specifically, beam rotates clockwise by angle $\psi$.

In general, the circular array mode zero ($m = 0$) gives an omnidirectional radiation pattern (*broadcast*), albeit with low directivity. The sum of all the modes results in a directional radiation pattern, with one main lobe (*uni-cast*), but now with higher directivity. Multi-mode circular antenna array also allows *multi-cast* transmission: by creating array excitation by summing up all the modes except zeroth mode, the final radiation pattern includes multiple lobes, and thus, antenna can transmit to different directions.

**Figure 1:**
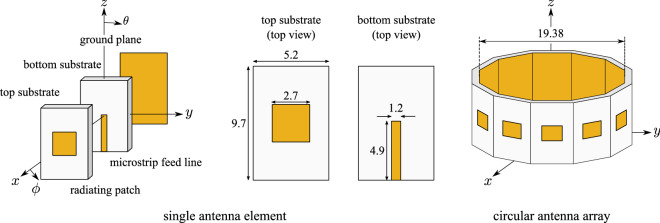
Structural configuration of the multilayer (double substrate) electromagnetically coupled, microstrip patch antenna element and the 12-element circular patch antenna array illustration: the dimensions of the antenna (in mm) are also depicted. Radiating rectangular patch with edge of 2.7 mm is placed in the centre of the top substrate of dimensions $5.2~\mathrm {mm} \times 9.7 ~\mathrm {mm} \times 0.5 ~\mathrm {mm}$. The bottom substrate has the same dimensions as the top, and here, the microstrip feed line with dimensions $4.9~\mathrm {mm} \times 1.2 ~\mathrm {mm}$ is placed. The circular antenna array, which lays on the *xy*-plane, has diameter of 19.38 mm ($1.8\lambda$): parameters $\theta$ and $\phi$ denotes the polar and azimuthal angle.

#### Circular patch antenna array design

**Figure 2:**
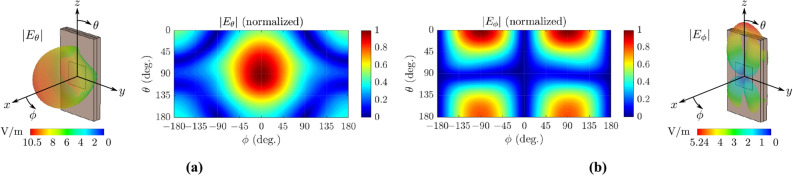
Simulated far-field radiation pattern of a single antenna element, which lays in the *zy*-plane versus polar (i.e., $\theta$) and azimuthal (i.e., $\phi$) angle: $E_{\theta }$ (**a**) and $E_{\phi }$ (**b**) denotes the electric field component in the $\mathbf {\theta }$ and $\mathbf {\phi }$ direction. $E_{\theta }$ component, which is vertical to the horizontal plane (i.e., *xy*-plane), is maximized at $\theta =90^{\circ }$ and $\phi =0^{\circ }$, thus, in the horizontal plane and specifically in front of the single antenna element: this component will lead to multimode operation in the circular antenna array geometry. $E_{\phi }$ component, which is tangential to the horizontal plane, is maximized at $\theta =0^{\circ }$, thus, in the vertical direction and specifically at the top of the single antenna element: this component will lead to the form of OAM-modes in the circular antenna array. Also, please note that where $E_\phi$ is maximized, $E_\theta$ is minimized, and vice-versa. The latter is the key-point that makes the proposed circular antenna bi-functional.

To realize the circular array, we use an electromagnetically coupled patch antenna as a single radiating element, which lays on a double layer substrate placed in *zy*-plane, as depicted in Fig. 1. The bottom layer is designed on Rogers RO4003 substrate ($\varepsilon _r = 3.38$, $\tan \delta = 0.0027$) while the top layer is designed on Taconic TLY-5A substrate ($\varepsilon _r = 2.17$, $\tan \delta = 0.0009$): each one of these has dimension of $5.2~\mathrm {mm} \times 9.7~\mathrm {mm} \times 0.5~\mathrm {mm}$. The antenna element is fed through a microstrip transmission line of length 4.9 mm and width of 1.2 mm, designed on the bottom substrate, with full ground plane. Please note that, there is a gap between the feeding mechanism and radiating patch, which helps in hosting additional radiating fields. The latter allows high directivity radiation not only along the $+x$-direction (as in microstrip patch antennas^23^), but also along the $+z$-directions, as it will be shown. Next, the microstrip line is fed by a 50 $\Omega$ lumped port when the antenna is simulated through full-wave electromagnetic simulation in CST Studio Suite, in order to operate at 28 GHz, and the optimized design parameters are presented in Fig. 1. The antenna element is also tested in terms of radiation pattern and the results are depicted in Fig. 2: the simulated total realized gain and radiation efficiency at that frequency of 5.74 dBi and $98\%$, respectively. Based on Fig. 2 it is observed that for the antenna element the far-field electric component $E_{\theta }$ (i.e., electric component in $\theta$ direction), which is vertical to the *xy*-plane (i.e., horizontal plane) is maximized at $\theta =90^{\circ }$ and $\phi =0^{\circ }$, i.e., in front of the antenna, leading to multimode transmission in the horizontal plane for the case of the circular antenna array. Also, the far-field electric component $E_{\phi }$ (i.e., electric component in $\phi$ direction), which is tangential to the *xy*-plane, is maximized at $\theta =0^{\circ }$, i.e., at the top of the antenna, leading to OAM transmission in the vertical direction for the case of the circular antenna array. Actually, where $E_\phi$ is maximized $E_\theta$ is minimized, and vice-versa. The benefit of this antenna radiation mechanism will be shown in the later section when same antenna will be used for multi-cast and OAM mode radiation in different directions. Also, please note that the transmitted wave in *x*-direction has the double magnitude to the transmitted wave in the *z*-direction (i.e., 10.5 V/m and 5.24 V/m, respectively), and thus, the transmitted power to these two directions is comparable. This property is crucial in the design of the circular antenna array, where the transmitted power in horizontal and vertical direction, under uni-/multi-cast and OAM modes, respectively, should be equivalent order of magnitude.

**Figure 3:**
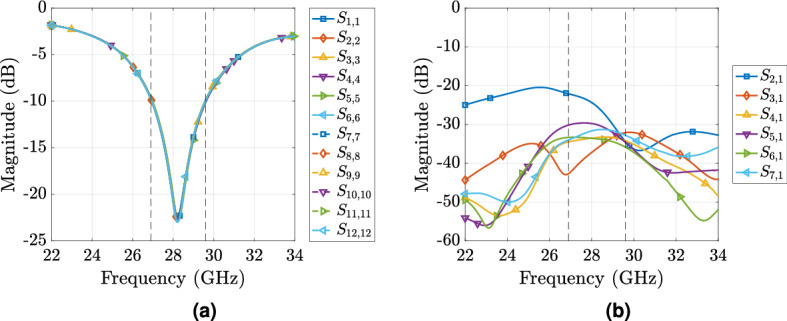
Simulated reflection coefficients relative to $50~\Omega$ (**a**) and mutual coupling (**b**) of the 12-element circular antenna array: analysis took place in CST Studio Suite and lumped ports of $50~\Omega$ were used to simultaneously feed all the antenna ports. For the mutual coupling analysis only 7 out of 12 elements were tested due to the cylindrical antenna array symmetry.

**Figure 4:**
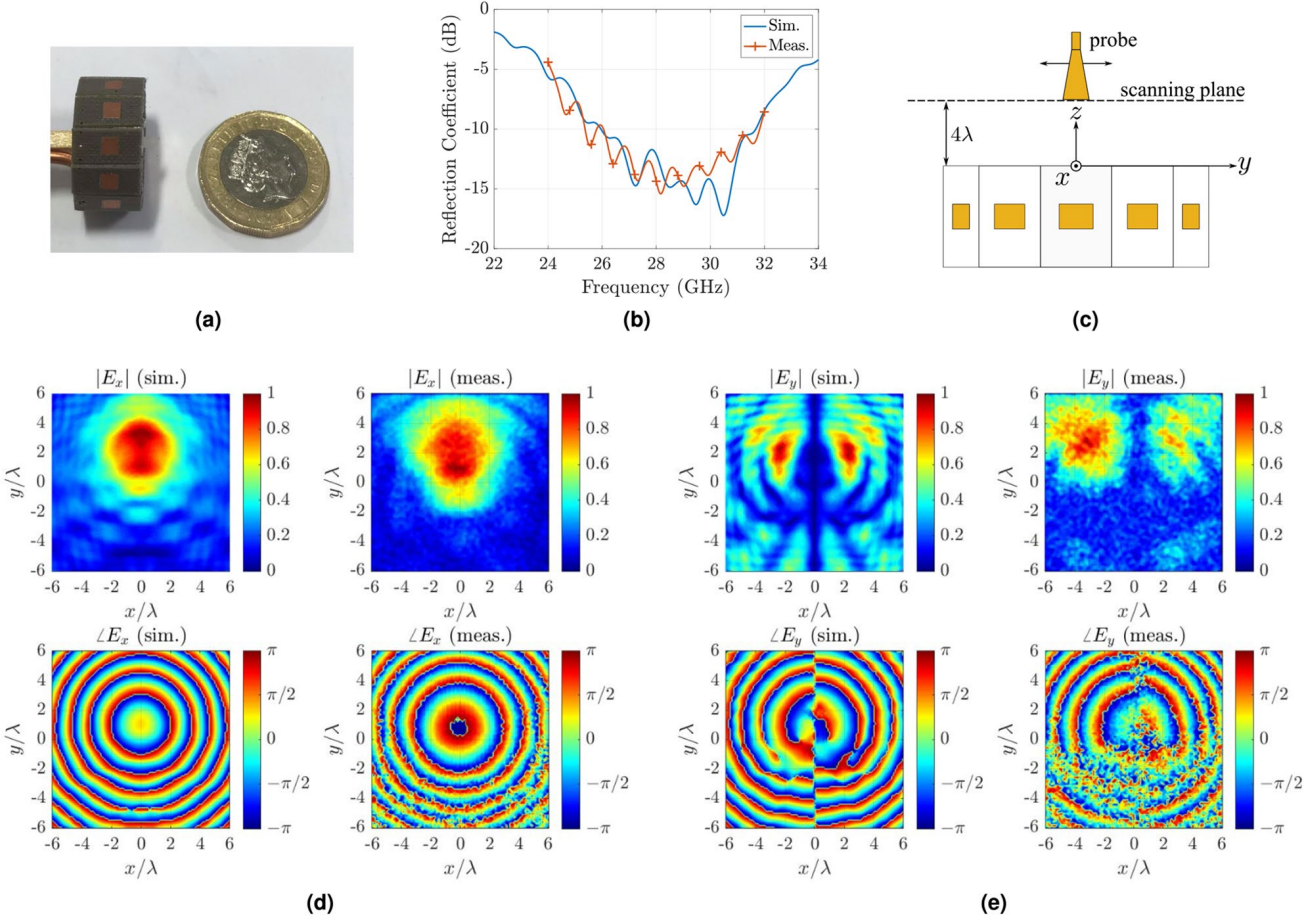
(**a**) The fabricated and tested circular antenna array: only a single element is fed by a $50~\Omega$ coaxial cable and all the other ports are open-circuited. (**b**) Comparison between simulated and measured reflection coefficient at the end of the coaxial cable: a good-agreement is observed. (**c**) The near-field measurement set-up: probe, which is connected to VNA measures the electric field (i.e., $E_x$ and $E_y$ component) when the antenna transmits at 28 GHz. Comparison between simulated and measured $E_x$-, $E_y$-field patterns in the form of normalized magnitude and phase: a good agreement is observed.

For the circular antenna array, 12 copies of the this antenna elements are vertically placed in a circular formation, as shown in Fig. 1 in the horizontal plane (i.e., in the *xy*-plane) and the obtained circular antenna array diameter is $d = 19.38$ mm (i.e., $1.8\lambda$ at 28 GHz). Simulated *S*-parameters of the circular array were estimated in CST Studio Suite when all antenna ports are fed by lumped ports of 50 $\Omega$ in order to test simultaneous array excitation and they are shown in Fig. 3. Reflection coefficient results relative to $50~\Omega$ depicted in Fig. 3a show that each antenna operates from 26.95 to 29.64 GHz ($9.5\%$ fractional bandwidth) with a |S_11_| lower than $-10$ dB. Please note that, due to cylindrical symmetry of the array based on (1), mutual coupling results against excitation of only 7 ports are shown in Fig. 3b. The worst cross coupling is between immediate neighbouring elements ($-24$ dB at 28 GHz) while it is below this value for the rest of the cases.

Next, the circular antenna array was fabricated and measured. During the fabrication process, the array was realized using a combination of separately fabricated patch antennas. Based on our lab facilities and given the small antenna dimensions, we managed to connect only one of the antenna array elements to a $50~\Omega$ coaxial cable and we left the other ports open-circuited, as depicted in Fig. 4a. Then, we connected the coaxial cable to a Vector Network Analyser (VNA) via a mini-SMP connector, and the reflection coefficient relative to $50~\Omega$ was measured and the results are depicted in Fig. 4b. In order to have a fair comparison between measured and simulated results, now the latter were estimated when only a single antenna element is fed thought a $50~\Omega$ coaxial cable and all the other ports are open-circuited. A good agreement is observed between the measured and simulated results and the antenna operates at 28 GHz.

After confirmation of antenna’s array impedance matching, near-field measurements were performed. Antenna array was placed at distance of $4\lambda$ from a probe, as depicted in Fig. 4c, which is connected to a VNA and the magnitude and phase of the electric component along *x*- (i.e., $E_x$) and *y*-direction (i.c., $E_y$) was measured when the antenna array transmits at 28 GHz. Results shown in Fig. 4d,e verify the simulated predictions; a clear agreement between measured and simulated fields can be observed from the magnitude and phase plots for both *x*- and *y*-polarization fields. After successful verification of the circular array operation same array is used to generate multi-cast and OAM modes that are discussed in the proceeding sections.

**Figure 5:**
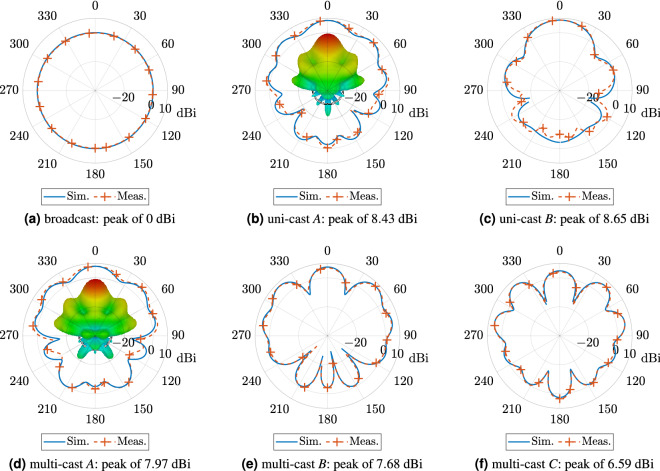
Comparison between simulated and measured realized gain in dB scaling for (**a**) general broadcast, (**b**) uni-cast *A*, (**c**) uni-cast *B*, (**d**) multi-cast *A*, (**e**) multi-cast *B*, and (**f**) multi-cast *C* with maximum of 0 dBi, 8.42 dBi, 8.65 dBi, 7.96 dBi , 7.68 dBi and 6.58 dBi, respectively: a good agreement is observed. Inset: simulated realized gain in 3D shape for the uni- and multi-cast *A* mode.

### Uni- and multi-cast transmission

The mode-mixing excitation of a circular array allows uni-cast and multi-cast radio transmission in addition to broadcast (i.e., $m=0$), and this is done by combining only some of the *m* modes based on (4). In this section, we show that the circular antenna array can generate, among others, two uni-cast and three multi-cast radio transmissions. The two uni-cast cases *A* and *B* are the result of the sum of the modes $m = 0, \pm 1, \ldots \pm 5$ and $m = 0, \pm 1, \pm 2, \pm 3$, respectively. Accordingly, the three multi-cast cases *A*, *B* and *C* are the result of mode mixing of $m = \pm 1, \pm 2, \ldots , \pm 5$, $m = \pm 3, \pm 4, \pm 5$, and $m = \pm 4, \pm 5$, respectively. The simulated results for the realized gain in the horizontal plane (i.e., *xy*-plane or $\theta =90^{\circ }$), estimated thought CST Studio Suite, are depicted in Fig. 5. First, the broadcast radiation pattern ($m=0$), where all the elements are excited by the same signal (i.e., magnitude 1 and phase 0), is depicted in Fig. 5a: the electric field is omni-directional, as expected in that case, with a maximum of 0 dBi. It is noted that the maximum directivity in that case is 4.86 dBi but occurs in the plane $\theta =15^{\circ }$ and not in the horizontal plane. Realized gain for the uni-cast modes *A* and *B* is depicted in Fig. 5b,c, respectively, and it has maxima of 8.43 dBi and 8.65 dBi, respectively. For the multi-cast cases, simulated results are shown in Fig. 5d–f, where the circular array is capable of data transmission in three dominant directions in the horizontal plane, i.e., $\phi = 0^{\circ }$. and $\phi = \pm {}45$ deg. Here the corresponding peak directivity at $\phi = 0 ^{\circ }$. decreases from 7.97 dBi (multi-cast case *A*) to 7.68 dBi (case *B*) and to 6.59 dBi (case *C*) at an expense of the radiated power from the array in multiple directions being equalized (i.e., at $\phi =0,\pm {}45$): latter is evident in Fig. 5f where almost equal power is radiated along three directions.

Broadcast and all cases of uni-cast and multi-cast are cross-verified through measurements, which were performed in anechoic environment. Specifically, the circular antenna array is placed in a turn table in the far-field anechoic chamber, as will be explained in Measurements section, and its radiated power at 28 GHz was measured along $360^{\circ }$ in the horizontal plane by a spectrum analyser through a receiver horn antenna. Next, the realized gain was estimated based on these measurements and on the *method three antennas*^24^. Measurement results are depicted in Fig. 5, alongside with the simulated results for comparison reasons: good agreement is observed. Based on these results, uni-cast case *B* presents the highest realized gain, thus it is a perfect candidate for directive transmission, whilst multi-cast case *B* and *C* can be utilized for simultaneous, multi-direction data transmission to multiple (i.e., three) clients, around the circular array, in the horizontal plane. Another significant contribution of the proposed antenna is that, radiation patterns, and thus, all the beams for the uni- and multi-cast transmission can be rotated by angle $\psi$, based on (5), and thus, the proposed, multi-mode antenna is *steerable*, providing $360^{\circ }$ rotation in the horizontal plane. Please note that the latter can be implemented by the dynamical change of the feed currents in terms of only the phase, by the application of various practical techniques, e.g., utilization of Rotman Lens and phase ramps geometries^18–20^.

**Figure 6:**
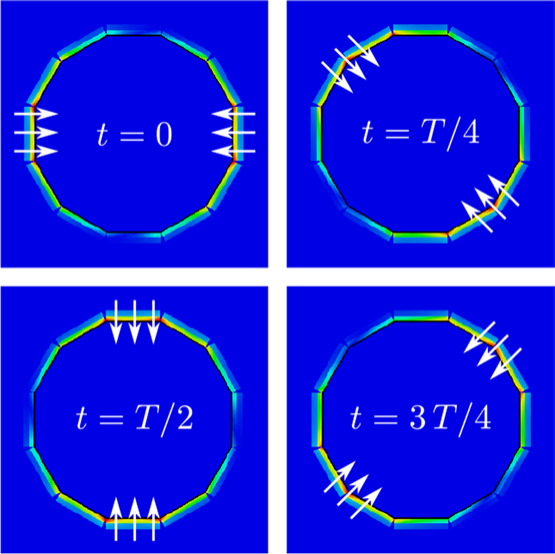
Simulated current distribution of the antenna array at different time phases, where *T* is the period, for the first mode (i.e., $\ell = +1$) at 28 GHz. Series of radiating copper edges tangential to the top plane of the circular antenna array, which are rotating versus time, leads to radiation in *z*-direction over the tangential to the horizontal plane $E_{\phi }$ electric component, which, in turn, leads to formation of OAM modes along *z*-axis: this operation mechanism is well-described in^25^.

### OAM mode transmission

Advantageously, the same circular antenna array in Figs. 1 and 4a can generate up to 15 OAM modes along *z*-direction (i.e., $\theta = 0^{\circ }$) when the array is placed in the reference horizontal plane (*xy*-plane). The generation of OAM modes is a consequence of radiation characteristics of the single antenna element, as previously described. Based on Fig. 2 it is evident that the antenna is capable of transmitting in both ways, well separated from each other. Specifically, in the horizontal plane (i.e., $\theta =90 ^{\circ }$) the dominant electric component is the $E_\theta$, which is normal to that plane, and thus, results vertical polarization and finally, multimodal operation, as has been shown. On the other hand, in the vertical direction (i.e., $\theta =0^{\circ }$) the dominant electric component is the $E_\phi$, which is tangential to the horizontal plane, and leads to OAM operation. Putting this statement into perspective, please, note the gap between the feeding line on Rogers RO4003 substrate and the radiating patch on Taconic TLY-5A substrate (Fig. 1): when the radiating elements are placed in circular array formation and then observe the antenna array from the top, we can observe a series of radiating copper edges, tangential to the array circle (Fig. 6), which are rotating versus time. These edges act as resonant slots and is primarily responsible for generation the OAM modes^25^. Mathematically, the excitation vector for the *N* element circular array is given by

6 $$\begin{aligned} w_i = e^{2j(i-1){\ell \pi }/{N}}, \end{aligned}$$

where $i = 1,2,\ldots N$ represents the antenna elements of the array and $\ell$ is the OAM mode number. The number of the antenna elements in the circular antenna array determines the number of the OAM modes that can be produced^25^. The radiation mechanism, which leads to OAM transmission is presented in Fig. 6. Specifically, it is depicted the simulated surface current distribution of the antenna array at different time phases (*T* is the period) for the first mode ($\ell =+1$). As the period increases by $45 ^{\circ }$, the current phase is shifting accordingly. Also, the current vectors have opposite direction, as described in^25^ for the same mode ($\ell =+1$), which finally, leads to OAM transmission.

**Figure 7:**
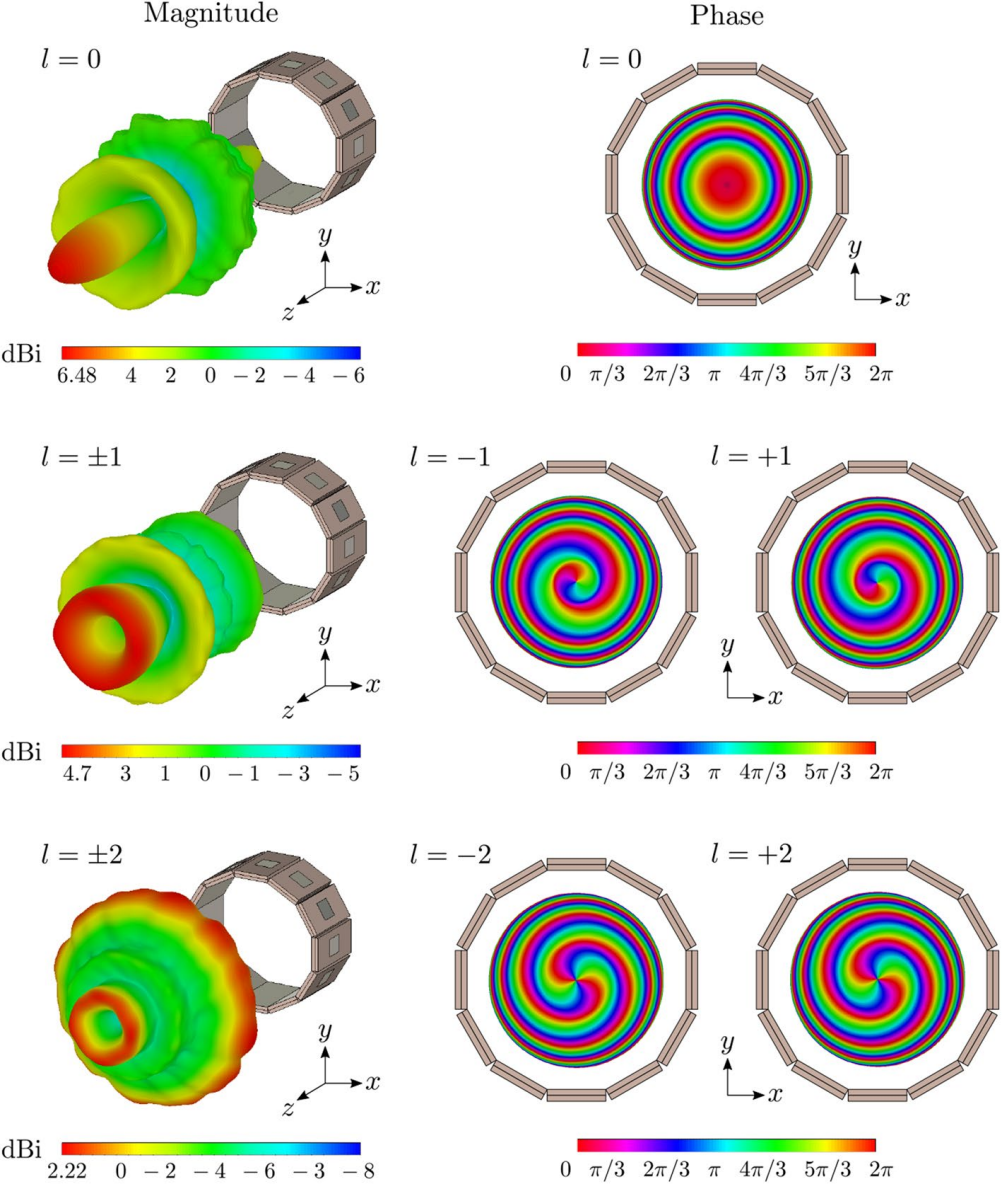
Simulated far-field realized gain patterns (magnitude and phase) of the OAM modes $l=0,\pm {}1,\pm {}2$ in dB scaling. Phase helical structures occurs for modes $l\pm {}1,\pm {}2$ with an optical vortex in the center, while for mode $l=0$, phase is not helical, but forms multiple disconnected concentric circular surfaces. The difference between positive and negative OAM modes in terms of phase is that, in the first case the phase increases clockwise, while in the second case counter-clockwise.

**Figure 8:**
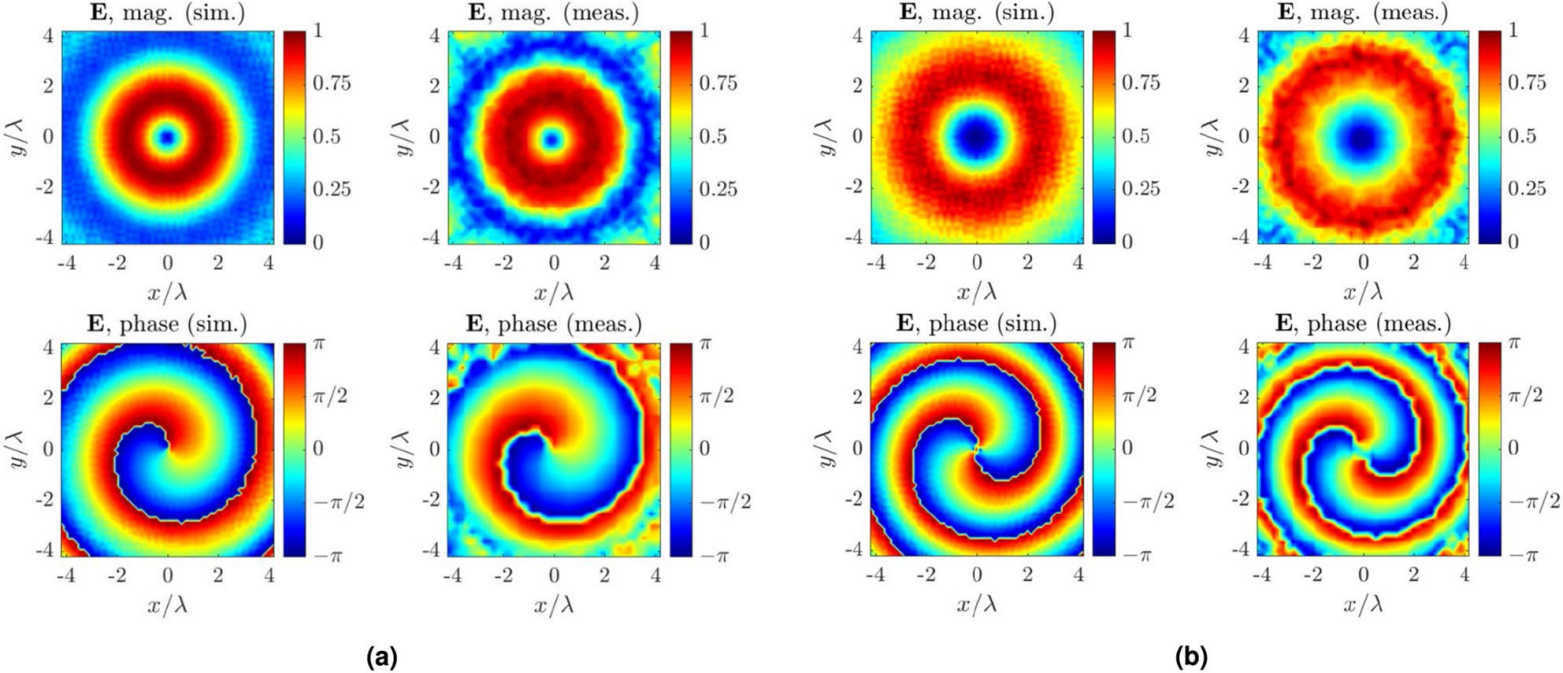
Comparison between simulated and measured near-field electric field patterns against OAM excitation for mode (**a**) $\ell = +1$ and (**b**) $\ell = +2$: a good agreement is observed.

Next, the circular antenna array was tested in terms of OAM transmission. Specifically, the realized gain was estimated and the results are depicted in Fig. 7. Although the proposed antenna is capable of producing up to 15 OAM modes, for the shake of simplicity, we present in this work only the $\ell = 0,\pm {}1,\pm {}2$ OAM modes. Firstly, excitation of mode $\ell = 0$ reveals directional radiation pattern of 6.48 dBi peak realised gain at $\theta =0 ^{\circ }$. The half-power beamwidth is $19.4 ^{\circ }$. The phase variation is constant around the $+z$-direction. Secondly, modes $\ell = \pm 1$ and $\ell = \pm 2$ are excited and vortices are revealed along the $+z$-direction, while single and double helical structures (from $0 ^{\circ }$ to $360 ^{\circ }$) are observed in the radiation pattern phase: the phase spiral is clockwise for $\ell = +1, +2$ while it is counter-clockwise for $\ell = -1 , -2$. For the $\ell = \pm 1$ the realized gain has a peak of 4.7 dBi, occurs at $\theta =18^{\circ }$ and has half-power beamwidth of $17.6 ^{\circ }$ Also, for the $\ell = \pm 2$ the peak realized gain is now 2.22 dBi at $18 ^{\circ }$ and has half-power beamwidth of $21.4 ^{\circ }$ Another interesting feature to observe is that the radiation pattern magnitude for mode excitation $\ell = \pm 2$ reveals quasi-broadcast transmission along *xy*-plane in addition to the OAM modes in the $+z$-direction, which provides an additional flexibility in terms of application scenarios to the presented circular array. The latter and the wider half-power beamwidth for $\ell = \pm 2$ lead to lower peak realized gain for this case compared to the $\ell = \pm 1$. Resultant animated field files against OAM mode excitations can be accessed from online repository: https://go.qub.ac.uk/mmWaveCircularArray. Also please note that, the phase response for a given $\ell$-th modes is responsible for spatial orthogonality, hence simultaneous data transmission using OAM modes is possible, as verified by literature^6,11–18^.

Based on the results presented in Figs. 5 and 7, for the uni/multi-cast and OAM modes, respectively, it is evident that the highest realized gain in the horizontal plane is 8.65 dBi for the uni-cast *A* mode, while the highest gain in the vertical direction is 6.48 dBi for the $\ell = 0$ OAM mode, thus, 2.2 dB less. The latter is in accordance with the radiation pattern of the single antenna element results presented in Fig. 2, where the radiated field in the vertical direction has the half magnitude (i.e., 5.24 V/m) of the radiated field in the horizontal plane (i.e., 10.5 V/m). Thus, based on the above analysis, the transmission in the horizontal plane (i.e., uni-/multi-cast modes) is comparable to the transmission in the vertical direction (i.e., OAM modes) in terms of radiating power.

OAM modes $\ell =1,2$ were verified through measurements in planar near-field anechoic chamber. The circular antenna array was placed at a distance $4\lambda$ from a scanning probe, operating at 28 GHz, which was placed along $+z$-direction when antenna was placed in the *xy*-plane. Figures 4c and 10a depicts the near-field measurement setup. Next, antenna array was set to transmit  at 28 GHz and the electric field was measured by the probe. Figure 8 illustrates the normalized measured results alongside with the simulated via CST Studio Suite. Please note that, the simulated helical phase was rotated by $180 ^{\circ }$ in a post-processing step for a fair comparison with the measured helical phase. Plots against $\ell = +1$ excitation in Fig. 8a show that the helical phase spiral is matched well with the simulated predictions while the magnitude is flattened around the vortex in $+z$-direction. Similarly, measured helical phase matches well with the simulated results for $\ell = +2$ excitation. All the above measured results verify the OAM function of the antenna array in the vertical direction.

**Figure 9:**
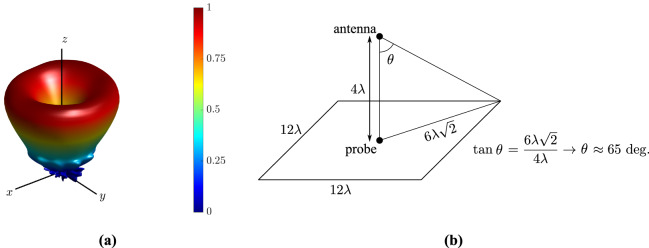
Normalized far-field radiation pattern (3D representation) of the circular antenna array, generated by the near-field measurements, estimated through the near- to far-field transformation (based on the *asymptotic evaluation* method), for the first OAM mode ($\ell =1$). Also presented the maximum angle that the normalized radiation pattern is valid, which is from $-65 ^{\circ }$ to $65 ^{\circ }$.

Finally, the far-field radiation pattern is estimated from the near field measurement results, through the near to far-field transformation, based on the *asymptotic evaluation* method, described in^24^, and the results (3D representation of the normalized magnitude in spherical coordinates) is depicted in Fig. 9a. Please note that for this estimation, probe scanned a region of $12\lambda$ by $12\lambda$ size lying at distance of $4\lambda$ from the antenna and thus, the maximum angle that the normalized radiation pattern in Fig. 9a is valid from $-65 ^{\circ }$ to $65 ^{\circ }$, as depicted and explained in Fig. 9b.

## Methods

### Simulation setup

The antenna was simulated in terms of reflection coefficient, mutual coupling and radiation pattern through full-wave electromagnetic analysis by using the commercial numerical solver CST Studio Suite. Specifically, the frequency domain solver (finite element method (FEM)) was used. Discrete, lumped ports at 50 ohm were applied as excitation. In order to estimate the radiation pattern for each mode, for the uni- and multi-cast and for the OAM transmission, the schematic representation of the antenna and the *AC, combine result simulation task* tool was used.

### Measurement setup

**Figure 10:**
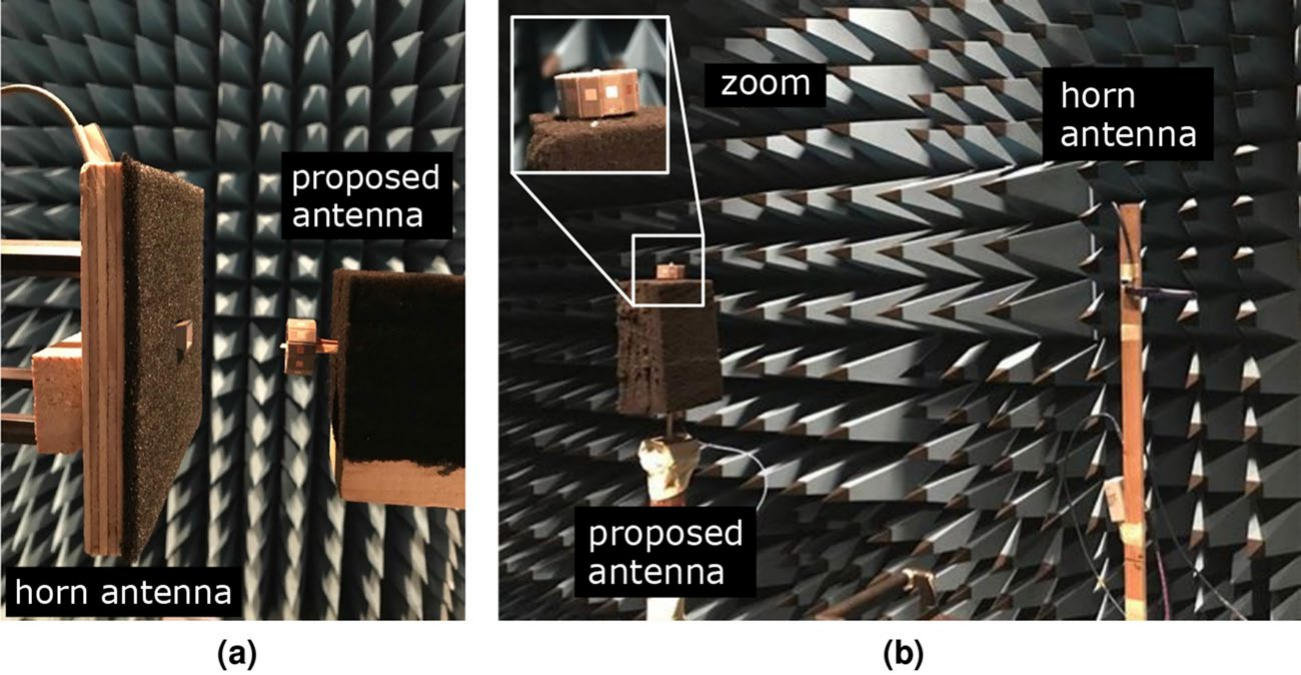
Measurement setup for the near- (**a**) and far-field (**b**) measurements.

The measurement setup for the near- and far-field is depicted in Figs. 4c and 10, respectively. For the near-field measurements the proposed antenna was placed at vertical distance of $4\lambda$ from a receiver probe at 28 GHz. The latter scanned a planar region with dimensions $12\lambda \times 12\lambda$, while capturing the transmitted electric field by the proposed antenna. The region was divided into $61\times {}61$ measurement points. For the cross-polarized component measurement (i.e., measurement of electric component $E_{y}$ in Fig. 4e) the probe was rotated by $90 ^{\circ }$. Please note that we performed near-field measurements with probe-antenna array under test distance of $8\lambda ,6\lambda ,4\lambda ,3\lambda$, trying to be within the near-field region and as closed to the antenna as possible, without affecting the measurement because of the presence of the probe. The reason was the right calibration of the phase: at high distance this was difficult, but at low distance the measurement data was affected. Thus, on each step we compared the simulated and measured results, and finally, we ended at distance of $4\lambda$, where a good agreement between simulated/measured results was observed. For the far-field measurement, the receiver horn antenna is placed at $100\lambda$ distance. Both antennas lie in the horizontal plane. Now, the proposed antenna rotates $360 ^{\circ }$ around its axis and the horn antenna captures the transmitted signal. Also, please note that the near- and far-field measurements were similarly performed: only one element of the antenna array was connected to a signal generator when all the other were open-circuited and then the array transmitted power, which was captured by the probe (near-field) or the horn antenna/receiver (far-field). The final measured results presented in this work in Figs. 5 and 8 were constructed in a post-processing stage where feed network and array calculations were used.

## Conclusion

This work presents a circular patch antenna array capable of generating uni- and multi-cast transmission along azimuth plane and, at the same time, a high number of OAM modes in elevation direction. The mode-based analysis is thoroughly investigated through fundamental principles and it is shown that mode-mixing of circular antenna array can result in various uni-cast and multi-cast transmissions. OAM mode excitations can result in several OAM modes transmission, where most of the latter show high level of spatial orthogonality. Antenna was fabricated via a conventional milling machine and tested in terms of reflection coefficient, near- and far-field radiation patter. Simulated results through full-wave electromagnetic analysis and measured results agree very well in all cases. Study around peak realized gain and comparison between all radiation modes in terms of radiated power reveals that the array radiation along horizontal plane and vertical axis is comparable, making the proposed antenna a viable practical solution. The array is perfect candidate for high spectral efficiency data transmission for 5G and beyond wireless applications. We envision that the array architecture with the specified radiation performance can be used in applications where on-demand azimuth and elevation coverage is desirable, e.g. in drone networks. One example of such scenario is the communication between drone and a base-station equipped with the circular antenna array where point-to-point link relies on OAM modes while uni- and multi-cast modes are for the communication between base-station (BS) antenna array and the user equipment. Another application is the communication between swarms of connected drones equipped with the circular array, where drone-to-drone communication is established along horizontal plane via uni- and multi-cast modes, while drone-to-BS communication along vertical axis uses OAM modes.
